# Supplementary figures and images for: MicroRNA-210-3p is transcriptionally upregulated by hypoxia induction and thus promoting EMT and chemoresistance in glioma cells
Source: PLoS One. 2021 Jul 1;16(7):e0253522. doi: 10.1371/journal.pone.0253522 (PMC8248614; doi:10.1371/journal.pone.0253522)

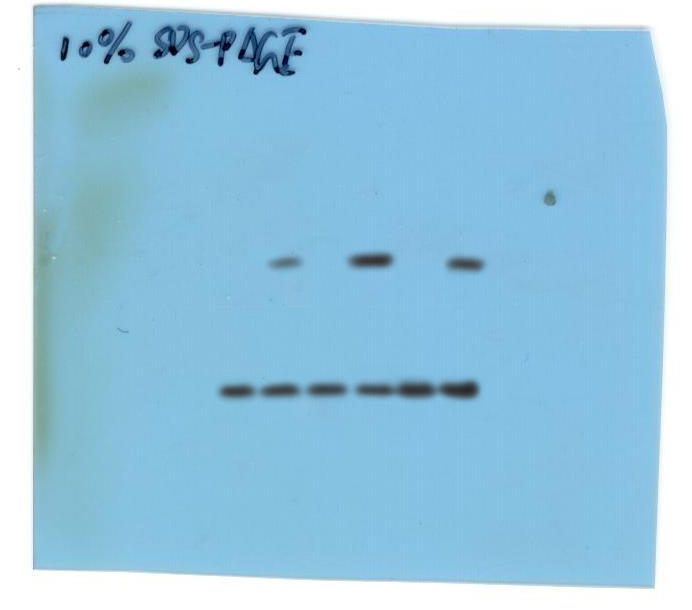

Supplement: S1 File — (ZIP) [file pone.0253522.s001.zip › supporting information/western images/figure 3c/WB.jpg]

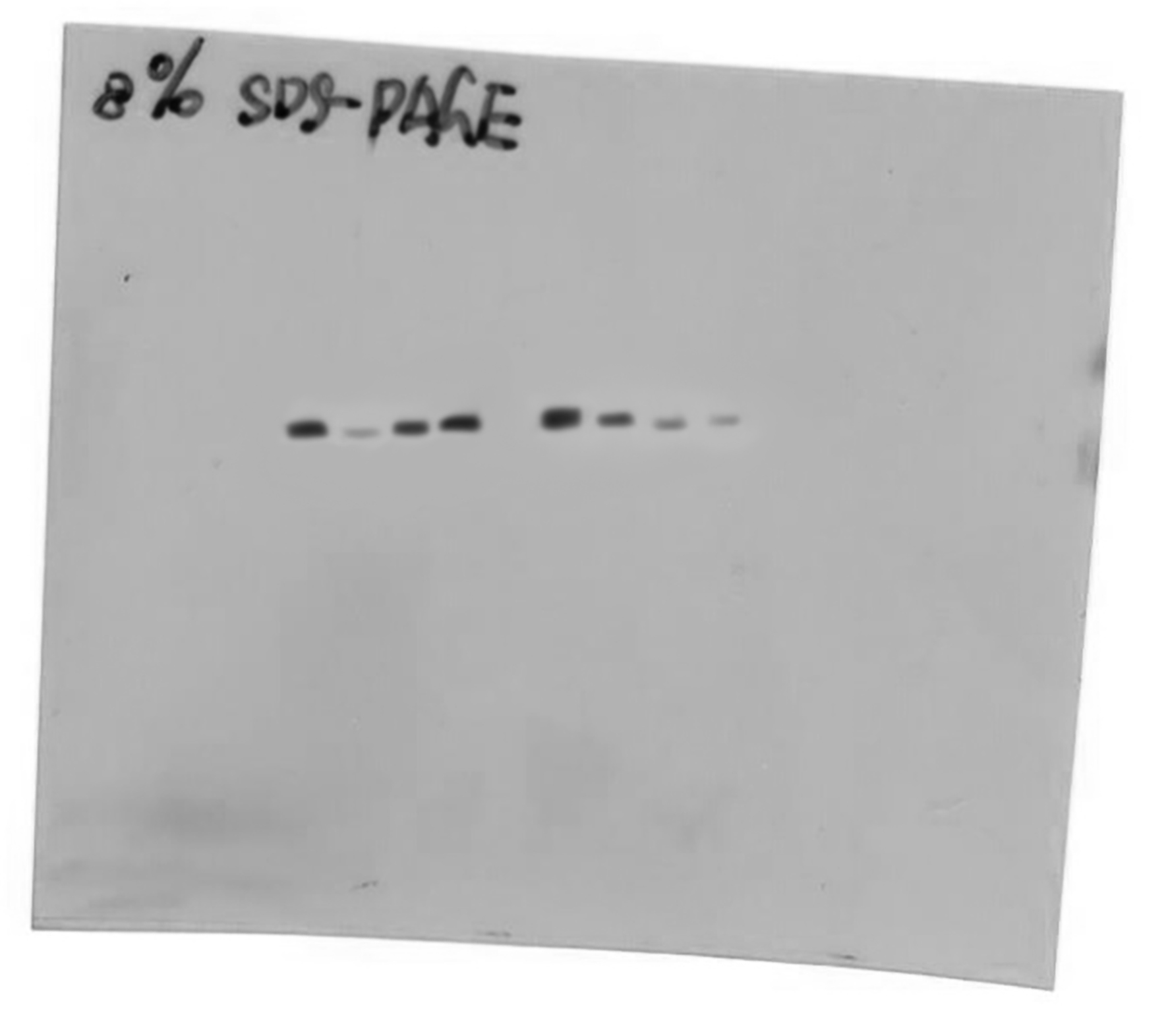

Supplement: S1 File — (ZIP) [file pone.0253522.s001.zip › supporting information/western images/figure 5a/E-cadherin.jpg]

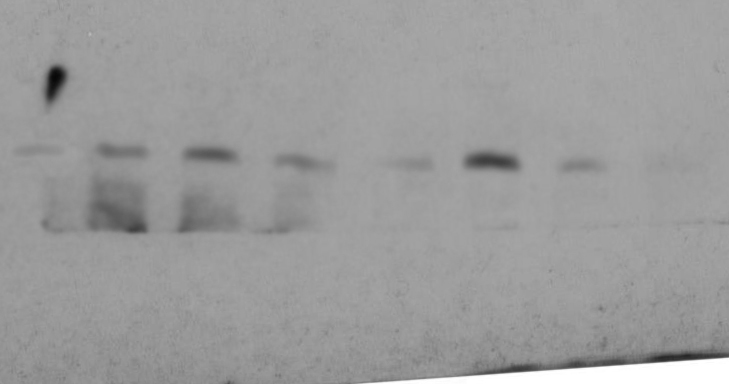

Supplement: S1 File — (ZIP) [file pone.0253522.s001.zip › supporting information/western images/figure 5a/N-cadherin.jpg]

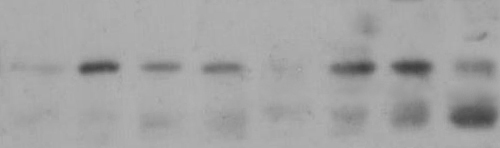

Supplement: S1 File — (ZIP) [file pone.0253522.s001.zip › supporting information/western images/figure 5a/Vimentin.jpg]

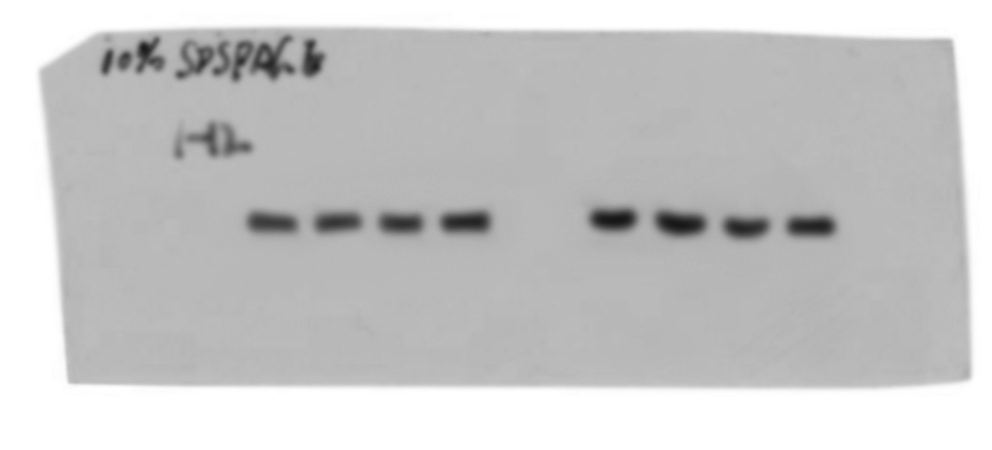

Supplement: S1 File — (ZIP) [file pone.0253522.s001.zip › supporting information/western images/figure 5a/actin.jpg]

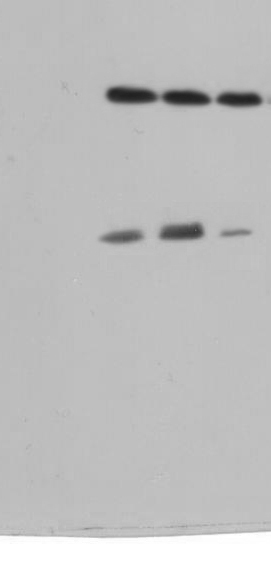

Supplement: S1 File — (ZIP) [file pone.0253522.s001.zip › supporting information/western images/figure 6b/WB.jpg]

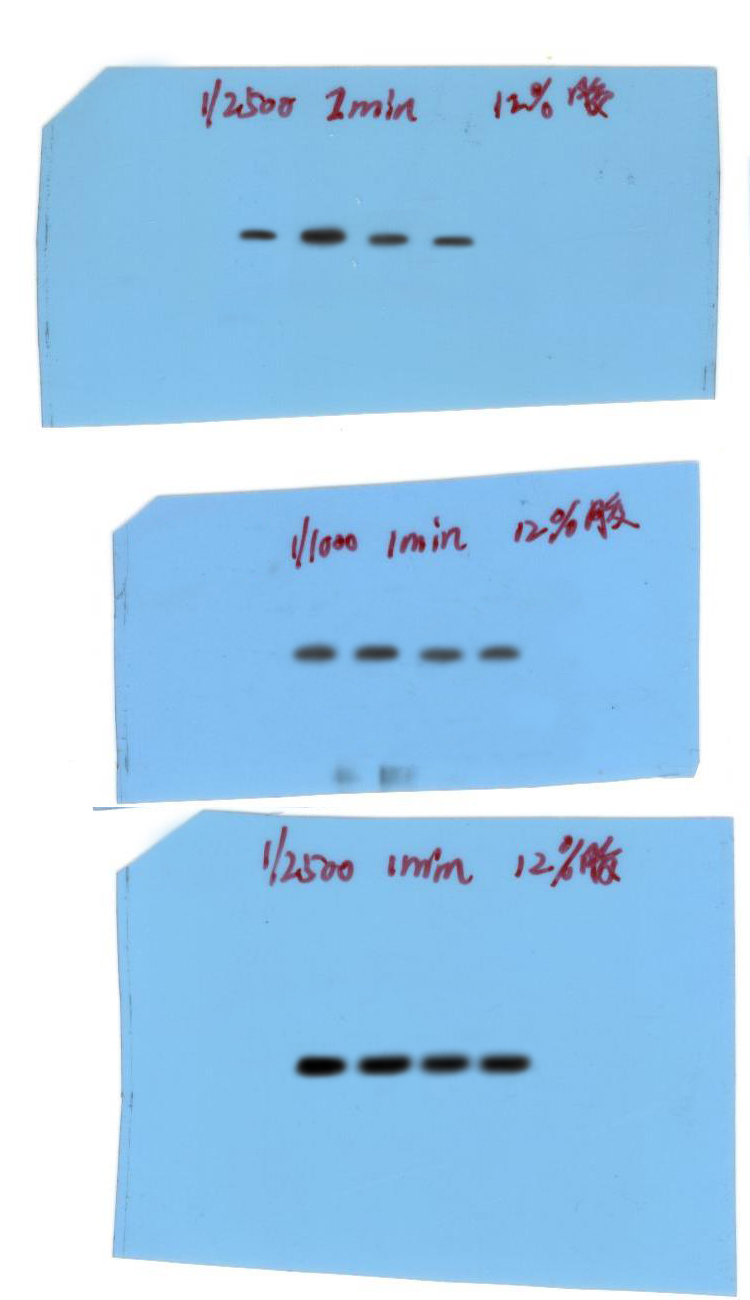

Supplement: S1 File — (ZIP) [file pone.0253522.s001.zip › supporting information/western images/figure 7c/7c.jpg]
