# Supplementary material for: MicroRNA-210-3p is transcriptionally upregulated by hypoxia induction and thus promoting EMT and chemoresistance in glioma cells
Source: PLoS One. 2021 Jul 1;16(7):e0253522. doi: 10.1371/journal.pone.0253522 (PMC8248614; doi:10.1371/journal.pone.0253522)
Supplement: Attachment — Submitted filename: response to reviewers.docx [file pone.0253522.s002.docx]

PONE-D-21-00617
MicroRNA-210-3p is transcriptionally upregulated by hypoxia induction and thus promoting EMT and chemoresistance in glioma cells
PLOS ONE

Dear Dr. Zhao,

Thank you for submitting your manuscript to PLOS ONE. After careful consideration, we feel that it has merit but does not fully meet PLOS ONE’s publication criteria as it currently stands. Therefore, we invite you to submit a revised version of the manuscript that addresses the points raised during the review process.
Before I send the manuscript out for review, there are number of points that I think need to be clarified.

• In the methods, what exactly does cells "were stored immediately to avoid being passaged for too many times" mean - how many times were they passaged?.

Answer：It has been modified as follows: “For each cell line, they were stored in liquid nitrogen before 3-5 passages.”
• The levels of hypoxia under various culture conditions need to be specified. Are the cultures actually anoxic? How are final O2 levels measured? How do they compare with O2 levels in situ, that is, within a tissue? This speaks to the physiological relevance of these studies.

Answer：The achieve of hypoxia condition was described in material and methods section as follows: “To achieve hypoxia, cells were maintained in a modular incubator chamber (Billups Rothenberg Inc., Del Mar, CA) containing 5% CO2, 95% N2 for 24 to 48 h. To achieve HIF-1α stabilization, 100 μM CoCl2 for 24 to 48 h to mimic hypoxia. “ All cells suffered from hypoxia is performed as described above.

Culture medium is anoxic. It was pre-stored in a modular incubator chamber (Billups Rothenberg Inc., Del Mar, CA) containing 5% CO2, 95% N2 for 24h to allow oxygen release.

Final O2 level is below 0.1% by following the instruction of modular incubator chamber. Medium is anoxic by pre-stored in hypoxia incubator for 24 h.

partial pressure of oxygen in situ is approximately 30 mmHg, which is high than that in O2＜0.1% condition.

• The use of CoCl2 treatment to mimic hypoxia seems problematic, not withstanding its convenience. Do the authors have comparative RNA SEQ analyses (for example) to establish how closely CoC2 treatment and hypoxia conditions resemble each other? This seems necessary given that "CoCl2 influences the transcription of distinct sets of genes that were not affected by low oxygen‐induced hypoxia, indicating that genes induced by both models of hypoxia do not overlap (Vengellur et al., 2005)." (from Muñoz‐Sánchez and Chánez‐Cárdenas, 2019).

Answer：The reason why cocl2 was used is that activation of HIF-1a by cocl2 was thought to be critical for the upregulation of miR-210 by hypoxia. Hif-1a is significantly upregulated by cocl2 treatment. We then employed echinomycin to blocked transcriptional activity of hif-1a, and expectedly, addition of echinomycin inhibited miR-210 in both hypoxia and cocl2 group, indicated that hif-1a which was both activated in hypoxia and cocl2 group was critical for miR-210, despite of other potential signaling.

• The authors should use the full name echinomycin rather than the non-standard abbreviation (EC)

Answer：It has been modified.

• and should note that "echinomycin strongly inhibits the activity of HIF‐1 under hypoxic conditions, and also interferes with the activity of other transcription factors. These results demonstrate the lack of specificity of this molecule. Moreover, it is demonstrated that echinomycin induces an increase in HIF‐1 activity under normoxic conditions, parallel to an increase in the expression of HIF‐1 target genes. (Vlaminck et al., 2007)

Answer：Thanks for the suggestion. In both hypoxia and cocl2 group, hif-1a was activated in hypoxia and cocl2 group, and this is the focus.

• The authors should present their studies in the context of related work on miRNAs and C6 glioma cells (He et al., 2020. "MiR 210 3p Inhibits Proliferation and Migration of C6 Cells by Targeting Iscu". Neurochemical research.)

Answer：Thanks for the suggestion, it has been added.

• and probably should have cited: Malzkorn et al. 2010) Identification and functional characterization of microRNAs involved in the malignant progression of gliomas. Brain Pathol 20:539–550

Answer：Thanks for the suggestion, it has been added.

Please submit your revised manuscript by Mar 06 2021 11:59PM. If you will need more time than this to complete your revisions, please reply to this message or contact the journal office at plosone@plos.org. When you're ready to submit your revision, log on to https://www.editorialmanager.com/pone/ and select the 'Submissions Needing Revision' folder to locate your manuscript file.

Please include the following items when submitting your revised manuscript:
• A rebuttal letter that responds to each point raised by the academic editor and reviewer(s). You should upload this letter as a separate file labeled 'Response to Reviewers'.
• A marked-up copy of your manuscript that highlights changes made to the original version. You should upload this as a separate file labeled 'Revised Manuscript with Track Changes'.
• An unmarked version of your revised paper without tracked changes. You should upload this as a separate file labeled 'Manuscript'.

If you would like to make changes to your financial disclosure, please include your updated statement in your cover letter. Guidelines for resubmitting your figure files are available below the reviewer comments at the end of this letter.

If applicable, we recommend that you deposit your laboratory protocols in protocols.io to enhance the reproducibility of your results. Protocols.io assigns your protocol its own identifier (DOI) so that it can be cited independently in the future. For instructions see: http://journals.plos.org/plosone/s/submission-guidelines#loc-laboratory-protocols

We look forward to receiving your revised manuscript.

Kind regards,

Michael Klymkowsky, Ph.D.
Academic Editor
PLOS ONE

Journal Requirements:
When submitting your revision, we need you to address these additional requirements.

1. Please ensure that your manuscript meets PLOS ONE's style requirements, including those for file naming. The PLOS ONE style templates can be found at

https://journals.plos.org/plosone/s/file?id=wjVg/PLOSOne_formatting_sample_main_body.pdf and

https://journals.plos.org/plosone/s/file?id=ba62/PLOSOne_formatting_sample_title_authors_affiliations.pdf

Answer: dear editor, the format of this manuscript has been modified following the two links.

2. PLOS ONE now requires that authors provide the original uncropped and unadjusted images underlying all blot or gel results reported in a submission’s figures or Supporting Information files. This policy and the journal’s other requirements for blot/gel reporting and figure preparation are described in detail at https://journals.plos.org/plosone/s/figures#loc-blot-and-gel-reporting-requirements and https://journals.plos.org/plosone/s/figures#loc-preparing-figures-from-image-files. When you submit your revised manuscript, please ensure that your figures adhere fully to these guidelines and provide the original underlying images for all blot or gel data reported in your submission. See the following link for instructions on providing the original image data: https://journals.plos.org/plosone/s/figures#loc-original-images-for-blots-and-gels.

In your cover letter, please note whether your blot/gel image data are in Supporting Information or posted at a public data repository, provide the repository URL if relevant, and provide specific details as to which raw blot/gel images, if any, are not available. Email us at plosone@plos.org if you have any questions.

 Answer: the raw data was supported in supporting information.

3. We note that you have included the phrase “data not shown” in your manuscript. Unfortunately, this does not meet our data sharing requirements. PLOS does not permit references to inaccessible data. We require that authors provide all relevant data within the paper, Supporting Information files, or in an acceptable, public repository. Please add a citation to support this phrase or upload the data that corresponds with these findings to a stable repository (such as Figshare or Dryad) and provide and URLs, DOIs, or accession numbers that may be used to access these data. Or, if the data are not a core part of the research being presented in your study, we ask that you remove the phrase that refers to these data.

Answer: dear editor, the “data not shown” is removed. My original meaning is that TGF-β release in supernatant was undetected.
