# Supplementary material for: MicroRNA-210-3p is transcriptionally upregulated by hypoxia induction and thus promoting EMT and chemoresistance in glioma cells
Source: PLoS One. 2021 Jul 1;16(7):e0253522. doi: 10.1371/journal.pone.0253522 (PMC8248614; doi:10.1371/journal.pone.0253522)
Supplement: Attachment — Submitted filename: response to reviewer.docx [file pone.0253522.s003.docx]

"Please respond to the reviewer's specific comments and outline those responses for me.."

The reviewer comments, in section 6 are:

"Reviewer #1: Most comments have been adequately addressed. Previous Comment 1 was directed at the specificity of EC. Many "specific inhibitors" are not so specific and if knock-down of the supposed target is not employed the authors should discuss briefly the limitations of EC (i.e., what is its specificity--IC50--for HIF and whether it is known to affect other enzymes; in order for the reader to have a full appreciation of the possible limitations and implications of using this methodology. As regards previous Comment 2, the authors should make sure that the train of thought and interpretation of the findings as presented in their response to the Reviewer's comment is clearly made to the reader in the manuscript.

Answer: Echinomycin (Quinomycin A) is potent small-molecule and cell-permeable inhibitor of hypoxia-inducible factor-1 (HIF-1) DNA-binding activity. In discussion section, we discussed the reason why echinomycin was employed instead of siRNA targeting to HIF-1 mRNA as a limitation of this manuscript. The methodology of this choice was explained to make it more clear for reader to have a full appreciation of this limitation. The modified section was described as follows (3^rd^ paragraph, discussion section):

Based on our results, it appears that HIF-1α induction via both hypoxia exposure and CoCl2 treatment transcriptionally upregulated miR-210-3p, revealing the specific regulatory mechanism of miR-210-3p under hypoxic conditions. To address our aim of revealing the effects of miR-210-3p on hypoxia-induced EMT and invasiveness in glioma cells, we treated hypoxia-exposed cells with echinomycin or introduced a miR-210-3p inhibitor (Figure 3B). We observed that both of these two treatments had similar effects on invasiveness, indicating that transcriptional induction of miR-210-3p may play critical roles in these processes. We also evaluated the effects of miR-210-3p on other malignant behaviors, including proliferation, colony formation and tumor formation; however, while echinomycin treatment clearly affected these phenomena, the miR-210-3p inhibitor had no effect, indicating that miR-210-3p is mainly involved in the regulation of invasiveness. Echinomycin, a potent small-molecule and cell-permeable inhibitor of hypoxia-inducible factor-1 (HIF-1) DNA-binding activity [33], was employed to inhibit HIF-1 transcriptional activity and thus to investigate the regulation of HIF-1α on miR-210-3p. HIF-1α functions as a transcriptional regulator and also interacts with proteins, or even long non-coding RNA and thus exerts multiple functions [34,35]. By considering this, echinomycin was employed instead of siRNA targeting to HIF-1 mRNA to specifically inhibit HIF-1α transcriptional activity without disturbing other functions.

All other points were sufficiently addressed."
